# FeS@BSA Nanoclusters to Enable H_2_S‐Amplified ROS‐Based Therapy with MRI Guidance

**DOI:** 10.1002/advs.201903512

**Published:** 2020-02-19

**Authors:** Congkun Xie, Dong Cen, Zhaohui Ren, Yifan Wang, Yongjun Wu, Xiang Li, Gaorong Han, Xiujun Cai

**Affiliations:** ^1^ State Key Laboratory of Silicon Materials School of Materials Science and Engineering Zhejiang University Hangzhou Zhejiang 310027 P. R. China; ^2^ Key Laboratory of Endoscopic Technique Research of Zhejiang Province Sir Run Run Shaw Hospital Zhejiang University Hangzhou Zhejiang 310016 P. R. China

**Keywords:** chemodynamic therapy, FeS@BSA nanoclusters, reactive oxygen species (ROS)‐based therapy, synergetic therapy, synergetic tumor treatment, tumor theranostics

## Abstract

Therapeutic systems to induce reactive oxygen species (ROS) have received tremendous success in the research of tumor theranostics, but suffered daunting challenges in limited efficacy originating from low presence of reactants and reaction kinetics within cancer cells. Here, ferrous sulfide‐embedded bovine serum albumin (FeS@BSA) nanoclusters, in an amorphous nature, are designed and synthesized via a self‐assembly approach. In acidic conditions, the nanoclusters degrade and simultaneously release H_2_S gas and Fe^2+^ ions. The in vitro study using Huh7 cancer cells reveals that Fe^2+^ released from FeS@BSA nanoclusters induces the toxic hydroxyl radical (·OH) effectively via the Fenton reaction. More interestingly, H_2_S gas released intracellularly presents the specific suppression effect to catalase activity of cancer cells, resulting in the promoted presence of H_2_O_2_ that facilitates the Fenton reaction of Fe^2+^ and consequently promotes ROS induction within the cells remarkably. After intravenous administration, the nanoclusters accumulate in the tumors of mice via the enhanced permeability and retention effect and present strong magnetic resonance imaging (MRI) signals. The findings confirm this therapeutic system can enable superior anti‐tumor performance with MRI guidance and negligible side effects. This study, therefore, offers an alternative gas‐amplified ROS‐based therapeutic platform for synergetic tumor treatment.

## Graphical abstract

Ferrous sulfide‐embedded bovine serum albumin (FeS@BSA) nanoclusters, in an amorphous nature, are synthesized via a self‐assembly approach. In acidic conditions, the nanoclusters degrade and simultaneously release H_2_S gas and Fe^2+^ ions. Fe^2+^ release effectively induces hydroxyl radical via the Fenton reaction, while intracellular H_2_S gas selectively suppresses catalase activity of cancer cells, enabling promoted reactive oxygen species induction and remarkable antitumor performance.

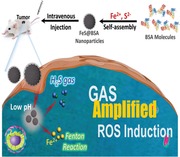

Therapeutic approaches mediated by reactive oxygen species (ROS) have received tremendous success in the exploration of cancer treatment.^[^[qv: 1]^]^ The elevated ROS content within cells leads to oxidative damage to lipids, proteins, and DNA, and consequently the apoptosis.^[^[qv: 2]^]^ Recent endeavor has been pursued on the exploration of therapeutic systems that may effectively response to the tumor microenvironment to trigger cell ferroptosis.^[^[qv: 3]^]^ Iron‐based nanoparticles, especially ones containing Fe^2+^ ions, have been demonstrated to induce cytotoxic hydroxyl radical (·OH) through Fenton reaction from hydrogen peroxide (H_2_O_2_) that presents in cancer cells, so‐called chemodynamic therapy (CDT).^[^[qv: 4]^]^ However, the reaction kinetics and the intracellular content of H_2_O_2_ are not adequate for maintaining continuous ROS induction, that restrains the efficacy of tumor treatment by CDT.^[^[qv: 5]^]^ Meanwhile, some intrinsic phenomena occurring in cancer cells have led to the resistance to CDT, and maintained the cancer progression.^[^[qv: 6]^]^ To resist the toxic ROS imposed exogenously or produced by their own metabolism, cancer cells may evolve a variety of response pathways. For example, glutathione is overexpressed in cancer cells (up to 10 × 10^−3^ m), which is an important pathway to protect cells from oxidative damage by ROS scavenging.^[^[qv: 7]^]^ Moreover, another important representative phenomenon is the production of excessive content of catalase (CAT).^[^[qv: 8]^]^ CAT is an essential antioxidant enzyme in cells, which plays a crucial role in maintaining the balance of intracellular ROS by decomposition of H_2_O_2_ and decreasing highly toxic ·OH production by intracellular iron through H_2_O_2_.^[^[qv: 9]^]^ A high expression of CAT has been found in various types of tumor cells specifically, which is primarily responsible for the failure of cancer therapy. The inhibition of the expression or activity of CAT has been recognized to be of fundamental importance for improving the efficacy of ROS‐based therapeutic approaches.^[^[qv: 10]^]^ However, an effective strategy for tackling this challenge remains lacking.

Recently, the use of specific gases for tumor inhibition, namely “gas therapy”, has emerged as a new type of effective therapeutic approach with minimal side effects.^[^[qv: 11]^]^ Three main gaseous molecules have been investigated to this purpose so far, including nitric oxide (NO), carbon monoxide (CO), and hydrogen sulfide (H_2_S). They are endogenous gaseous signaling molecules presented in mammals that play an important role in various physiological and pathophysiological processes.^[^[qv: 12]^]^ In fact, these gases have been used for treating diseases, in addition to cancer, ranging from inflammatory, nervous, and cardiovascular diseases.^[^[qv: 13]^]^ Since the discovery of NO as a gasotransmitter in human body by the Nobel Prize winner in 1998, H_2_S was the third gaseous transmitter presented in the human body.^[^[qv: 14]^]^ It regulates the functions of various enzymes in cells, mainly by the modification of cysteine residues to form protein persulfides called S‐sulfhydration.^[^[qv: 15]^]^ Previous studies have revealed that high concentration of exogenous H_2_S may induce the specific inhibition of cancer cells via cellular cycle arrest, miRNA regulation, mitochondrial damage, or uncontrolled intracellular acidification owing to the differences in metabolism and signaling pathway between cancer and normal cells.^[^[qv: 16]^]^ More importantly, the recent exploration uncovered that H_2_S can regulate CAT activity in some types of plant cells and bacteria, and this effect can be highly varied depending on cell types.^[^[qv: 17]^]^ Inspired by this phenomenon, H_2_S is expected to serve as a potential gaseous inhibitor to the CAT activity in certain cancer cells.

Therefore, in this study, Fe^2+^ and S^2−^ ions, which can serve as the donor of ROS and H_2_S gas, respectively, in the acid microenvironment containing H_2_O_2_, were diffused into bovine serum albumin (BSA) nanoparticles to form ferrous sulfide‐embedded BSA (FeS@BSA) nanoclusters with tens of nanometers in dimension through a self‐assembly approach (**Figure** **1**). BSA was chosen not only as the carrier matrix to ensure the colloid stability and biocompatibility, but also as the protector for FeS compound from oxidation in the physiological environment. The findings revealed that, owing to its amorphous nature, FeS@BSA nanoclusters may trigger rapid Fenton reaction to induce toxic ·OH, and release H_2_S effectively within Huh7 cancer cells. After intravenous injection to the mice, the nanoclusters accumulated in tumor site through the enhanced permeability and retention (EPR) effect and presented strong on‐site MRI signal. More interestingly, the intracellular H_2_S induced effective inhibition to the CAT activity specifically in the Huh7 cancer cells, and in turn promoted the intracellular content of H_2_O_2_ and amplified the subsequent Fenton reaction for toxic ROS production. In consequence, both in vitro and in vivo anti‐tumor efficacy were enhanced by a large magnitude, while negligible side effects were observed. This gas‐amplified ROS‐based therapeutic platform has, therefore, offered another conceptual strategy for synergetic tumor treatment.

**Figure 1:**
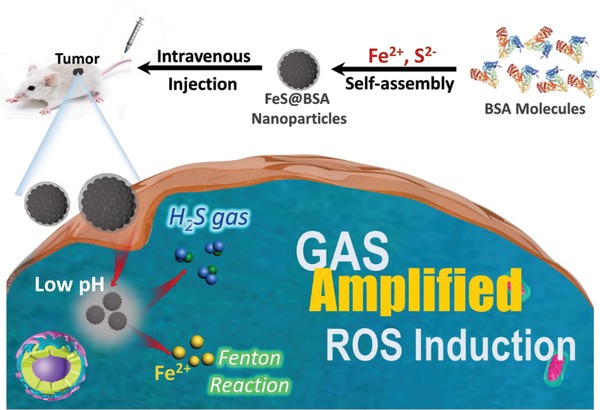
Schematic illustration of the synthesis and therapeutic process of FeS@BSA nanoclusters.

As examined by scanning electron microscopy and transmission electron microscopy, the FeS@BSA obtained exhibits a uniform spherical morphology with uniform dimension of ≈50 nm (**Figure** **2**a). Approximately 4 nm FeS nanodots with dark contrast are embedded in the BSA matrix, forming a nanocluster structure. The hydrodynamic size in solutions including pure water, phosphate‐buffered saline (PBS), NaCl solution, and Dulbecco's modified Eagle's medium remains ≈50 nm with good stability (Figure 2b; Figure S1, Supporting Information). The X‐ray diffraction patterns of FeS@BSA nanoclusters and BSA particles present no crystalline peaks (Figure 2c), reflecting that FeS nanodots embedded are amorphous phase. Meanwhile, the chemical composition of FeS@BSA examined by elemental mapping confirms that the nanoclusters consist of N, Fe, and S elements, as expected (Figure 2d). The UV–vis spectrum confirms the coexistence of BSA and FeS compounds in the nanoclusters (Figure S2, Supporting Information). The content of FeS in FeS@BSA is quantified to be 6.5% by using inductively coupled plasma mass spectrometry (ICP‐MS), which is almost equal to the feed ratio in FeS@BSA synthesis, indicating that nearly all Fe^2+^ added were absorbed on BSA molecules. It is known that amorphous iron nanoparticles present remarkable advantages compared to the corresponding nanocrystalline particles in triggering Fenton reaction due to their enhanced ionization and release of ferrous ions in acidic environment.^[^[qv: 18]^]^ Since BSA molecules were used as template agent for the nanocluster formation, the conformation change of BSA molecules is of crucial importance in determining the morphology and structure of the nanoparticles. As revealed by the circular dichroism (CD) spectra, the CD curves of BSA‐Fe^2+^ and FeS@BSA solutions are similar to that of the pure BSA solution, illustrating that the secondary structure of BSA remained unchanged during this synthesis (Figure S3, Supporting Information). The stretched secondary structure of BSA molecules may inhibit the formation of small nanodots with its molecular network due to steric effect.^[^[qv: 19]^]^ Meanwhile, FeS@BSA can remain in the same black color for 3 days when stored in 4 °C, and in contrast, the pure FeS nanoparticle solution became clear only after 2 h (Figure S4a, Supporting Information). The iron valence state of FeS@BSA during the storage was investigated by X‐ray photoelectron spectroscopy (Figure S4b, Supporting Information). The Fe 2p_3/2_ binding energy of 712.2 eV of as‐prepared FeS@BSA nanoclusters corresponds to pure FeS. Approximately 3.0%, ≈3.9%, and ≈14.0% FeS are oxidized to Fe_3_O_4_ on the surface of nanoclusters after being stored for 1, 2, and 3 days, respectively. In addition, FeS@BSA solution remains in a stable manner without clear color variation even after freezing and unfreezing procedures (Figure S4c, Supporting Information). This phenomenon indicates that BSA can effectively prevent the unexpected oxidation of FeS and maintain the particle stability in the solution.

**Figure 2:**
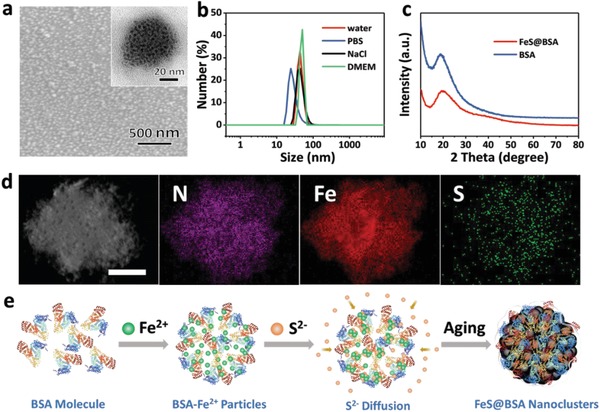
Characteristics of FeS@BSA nanoclusters. a) Scanning electronic microscopy image of FeS@BSA nanoclusters. Inset: high‐resolution transmission electron microscopy image. b) Hydrodynamic size of FeS@BSA nanoclusters in different solutions. c) X‐ray diffraction pattern of FeS@BSA nanoclusters and BSA particles. d) Elemental mapping of FeS@BSA nanoclusters. Scale bar is 20 nm. e) Schematic illustration of the formation of FeS@BSA nanoclusters during synthesis.

FeS@BSA nanoclusters were synthesized via a self‐assembly approach with aid of BSA molecules. BSA may initially absorb metal ions including Cu^2+^, Zn^2+^, and Fe^2+^, owing to its abundant functional groups, such as —NH_2_, —COOH, and —SH.^[^[qv: 20]^]^ As demonstrated in Figure 2e, when FeCl_2_ was added into BSA solution, Fe^2+^ ions bind to the BSA molecules and then form BSA‐Fe^2+^ particles. The color of particle solution remains unchanged. When Na_2_S was subsequently introduced to the reaction system, S^2−^ ions diffuse into the BSA‐Fe^2+^ framework, triggering the nucleation and formation of FeS within BSA matrix, and in consequence, the solution turns black (Figure S5, Supporting Information). After aging for 12 h and dialysis to remove excessive ions and unreactive reagents, amorphous FeS@BSA clusters formed.

In general, ferroptosis‐based therapeutic approach causes cancer cell death by generating ·OH with the presence of intracellular H_2_O_2_. In this study, 1,3‐diphenylisobenzofuran (DPBF) was used as an indicator to evaluate ·OH production of FeS@BSA in solutions with varied pH and H_2_O_2_ concentrations. Without FeS@BSA nanoclusters, the UV–vis spectrum of DPBF solution with pH of 6 and 200 × 10^−6^ m H_2_O_2_ remained unchanged, indicating its stability (Figure S6, Supporting Information). However, when FeS@BSA nanoclusters containing 2.5 µg mL^−1^ of FeS were added (buffer of pH = 7), the DPBF degraded clearly, and higher concentration of H_2_O_2_ induced more rapid DPBF degradation (**Figure** **3**a). The electron spin resonance was used to examine the radicals formed in the solution. Compared to pure H_2_O_2_ solution, typical peaks with ratio of 1:2:2:1 were observed when FeS@BSA was added in the H_2_O_2_ solution, indicating the formation of ·OH (Figure 3b). It is clear that higher content of ·OH is induced due to the Fenton reaction between FeS@BSA nanoclusters and H_2_O_2_ solutions with increased concentration. DPBF can be degraded completely within 10 min at a low concentration of FeS@BSA (2.5 µg mL^−1^ of FeS) and H_2_O_2_ (150 × 10^−6^ m), implying that FeS@BSA is a Fenton reagent with superior ability for ·OH induction. Meanwhile, it was found that higher FeS@BSA concentration and lower solution pH may also induce more rapid ·OH formation (Figure S7, Supporting Information), as expected.

**Figure 3:**
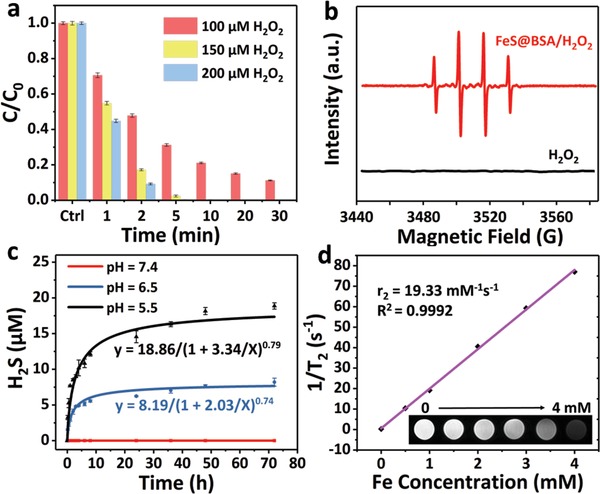
Intrinsic properties of FeS@BSA nanoclusters. a) Hydroxyl radical production of FeS@BSA nanoclusters (containing 2.5 µg mL^−1^ FeS) in DPBF solutions (pH = 7) with different concentration of H_2_O_2_ (*n* = 3, mean ± SD). b) Electron spin resonance spectra of FeS@BSA/H_2_O_2_ and pure H_2_O_2_ solutions with DMPO as the spin trap. c) H_2_S releasing profile in FeS@BSA solutions with different pH of 7.4, 6.5, and 5.5 (*n* = 3, mean ± SD, data fitting with Logistic5). d) The T2‐weighted MRI signals of FeS@BSA solutions with different concentrations (Fe concentration: 0.5 × 10^−3^, 1 × 10^−3^, 2 × 10^−3^, 3 × 10^−3^, and 4 × 10^−3^ m).

To explore the mechanism in the strong ROS induction by FeS@BSA nanoclusters in H_2_O_2_ solution, crystalline FeS particles were synthesized as a comparison (Figure S8a,b, Supporting Information). When the concentrations of iron ion and H_2_O_2_ were maintained at the same level, the degradation of DPBF in FeS@BSA solution was significantly accelerated compared to that with crystalline FeS solution (Figure S8c,d, Supporting Information). It is, therefore, considered that the amorphous nature of FeS@BSA nanoclusters enables the stronger capability of Fe^2+^ ions to react with H_2_O_2_ molecules, and facilitates the induction of ROS by a considerable magnitude. It is also noteworthy that the unique pH‐ and H_2_O_2_‐dependent ROS induction of FeS@BSA nanoclusters may potentially endow this platform with specified therapeutic properties by taking advantage of mild acidity and overproduced H_2_O_2_ in the tumor microenvironment.

FeS@BSA nanoclusters were dispersed and immersed in PBS with varied pH for different period of time. As revealed in Figure 3c, no H_2_S release was detected in the solution with a pH of 7.4. In contrast, clear H_2_S gas release of ≈10 × 10^−6^ m was observed in the solution with pH of 6.5 after 72 h, and the content of H_2_S released reached as high as ≈20 × 10^−6^ m when the solution pH was further reduced to 5.5. Higher concentration of FeS@BSA nanoclusters induced more rapid H_2_S release, as expected (Figure S9, Supporting Information). The morphology of FeS@BSA clusters did not present clear variation (Figure S10, Supporting Information). More importantly, owing to the shielding effect of BSA matrix, the reaction process of nanoclusters in the acid solution presents in a lasting manner. Sustained H_2_S release has been considered to be favorable for the tumor treatment compared with burst release.^[^[qv: 21]^]^ It is noteworthy that the microenvironment of tumor tissue (pH ≈6.5) is more acidic than normal tissues, while lysosomes are at an even lower pH of 5.5. The pH‐dependent release of H_2_S gas from FeS@BSA nanoclusters, due to its ionization in an acidic condition, is highly demanded in responding the specific tumor microenvironment in the therapeutic progress. It is noteworthy that most H_2_S donors including 1,2‐dithiole‐3‐thiones and GYY4137 produce by‐products when releasing H_2_S.^[16c]^ In many cases, their by‐products have not been well identified, and biological activities of these by‐products are unclear. There are only two functional factors of H_2_S and Fe^2+^ ions releasing from FeS@BSA in acidic environment without any additional by‐products. Therefore, FeS@BSA has been considered a preferred green donor of H_2_S.

Albumin‐based nanocomplexes have shown great potential for biomedical imaging and are extensively applied in fluorescence imaging, magnetic resonance imaging (MRI), positron emission tomography, and photoacoustic imaging.^[^[qv: 22]^]^ In this study, T2‐weighted MR images of FeS@BSA samples with controlled Fe concentrations were obtained using a 3.0 T clinical MR scanner. As expected, a typical concentration‐dependent darkening effect was observed (inset of Figure 3d). The T2 relaxation rates (1/T2) presented in a linear correlation against Fe concentration, and the calculated relaxation rate (r2) value was 19.33 × 10^−3^ m ^–1^ s^–1^, indicating that the FeS@BSA nanoclusters can also potentially serve as an effective MRI contrast agent.

When FeS@BSA nanoclusters (0–20 µg mL^−1^) were incubated with hepatocytes WRL‐68 cells in neutral environment for 24 and 48 h, the cell viability remained at a similar magnitude compared to the blank control, indicating that the samples do not present visible negative effect to the normal cells (**Figure** **4**a). Subsequently, the in vitro study using hepatocellular carcinoma (Huh7) cells was carried out to evaluate the antitumor properties of FeS@BSA nanoclusters. After incubation for 24 h, FeS@BSA presented clear toxicity to Huh7 cells under a neutral condition, and higher sample concentration induced more rapid killing phenomenon to the cancer cells (Figure 4b). More interestingly, when the incubation environment was varied from neutral to mildly acidic condition (pH = 6), the cell viability was substantially suppressed. In addition, when 100 × 10^−6^ m H_2_O_2_, which is a nontoxic dose to Huh7 cells (Figure S11, Supporting Information), was added to the culture medium, the cell‐killing effect was further improved. It is known that the cell‐killing effect is highly dependent on the ROS‐induced intracellularly by the nanoclusters. Subsequently, DCFH‐DA, which can be oxidized into fluorescent DCF by ·OH, was used as a probe to detect the presence of intracellular ·OH induced by FeS@BSA (Figure 4c). It was clear that no fluorescence was presented in the untreated cancer cells. In contrast, green fluorescence was observed when incubating Huh7 cells with FeS@BSA nanoclusters, and this fluorescence was dramatically enhanced under an acidic culturing condition, indicating a higher magnitude of ·OH production was induced. Meanwhile, the intracellular release of H_2_S was evaluated by FeS@BSA using a H_2_S‐specific fluorescence probe WSP‐1 (Figure 4d). Cells in the blank control group showed no detectable H_2_S, while clear fluorescence can be observed when incubating with FeS@BSA nanoclusters. In an acidic condition, high degree of H_2_S presented intracellularly, indicated by the enhanced fluorescence. This finding reflects that FeS@BSA nanoclusters, after internalized by cancer cells, may induce both ROS and H_2_S spontaneously owing to the acidic endosomes. The microenvironment with a lower pH facilitates the intracellular production of ·OH as well as H_2_S effectively, inducing enhanced killing effect to cancer cells.

**Figure 4:**
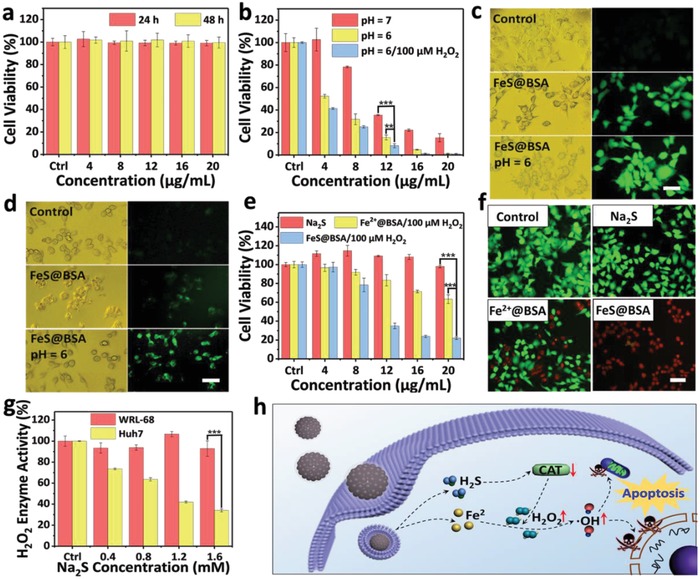
In vitro antitumor properties of FeS@BSA nanoclusters. a) Cytocompatibility of FeS@BSA nanoclusters to normal hepatocytes WRL‐68 (*n* = 3, mean ± SD). b) Viability of hepatoma carcinoma cell Huh7 cultured with FeS@BSA solutions with different concentrations and stated conditions after 24 h (*n* = 3, mean ± SD). c) Fluorescent images of Huh7 cells stained with DCFH‐DA for detecting intracellular ROS. Cells were incubated with 20 µg mL^−1^ of FeS@BSA in neutral and acidic (pH = 6) medium, respectively, for 6 h for ROS detection. The cells without treatment of FeS@BSA were used as the blank control. Scale bar is 20 µm. d) Intracellular H_2_S detection using WSP‐1 as a probe after cultured in medium (pH = 7.4 and 6.0, respectively) containing 20 µg mL^−1^ of FeS@BSA for 6 h, without treatment of FeS@BSA as a control. Scale bar is 20 µm. e) Viability of hepatoma carcinoma cell Huh7 cultured with Na_2_S, Fe^2+^@BSA, and FeS@BSA solutions at different concentrations after 24 h (*n* = 3, mean ± SD). f) Live/dead staining of Huh7 with calcein‐AM/propidium iodide after incubation with PBS, Na_2_S, Fe^2+^@BSA, and FeS@BSA for 24 h. Scale bar is 20 µm. g) Effect of H_2_S (Na_2_S as the donor) on H_2_O_2_ enzyme activity of WRL‐68 and Huh7 cells. 2 mL of the cell suspension (1 × 10^6^ mL^−1^) was treated with Na_2_S solutions at different concentrations for 30 min. H_2_O_2_ enzyme activities were determined by catalase assay kit according to its protocol (*n* = 3, mean ± SD). h) Schematic illustration of synergistic therapeutic mechanism of FeS@BSA nanoclusters. ***p < 0.001, **p < 0.01, and *p < 0.05.

In order to uncover the synergetic mechanism of H_2_S and ·OH induced by FeS@BSA nanoclusters in the enhanced cell‐killing phenomenon observed, the Fe^2+^@BSA nanoparticles, as a comparison, were synthesized following an identical procedure of FeS@BSA nanoclusters, but without the addition of Na_2_S. The cell‐killing effect of Na_2_S, Fe^2+^@BSA, and FeS@BSA under neutral condition was examined when the concentrations of S^2−^ in Na_2_S and Fe^2+^ in Fe^2+^@BSA were maintained at an equivalent level to FeS@BSA nanoclusters (Figure 4e). After 24 h, no clear variation in the viability was observed to the Huh 7 cells incubated with Na_2_S within the concentration range from 0.1 × 10^−3^ to 0.5 × 10^−3^ m, implying that H_2_S gas induced by Na_2_S did not induce cell‐killing effect directly. Meanwhile, FeS@BSA nanoclusters showed significantly enhanced cell‐killing effect compared to that of Fe^2+^@BSA particles, which agrees well to the images of live/dead cell staining (Figure 4f), owing to the intracellular corelease of Fe^2+^ and H_2_S after being internalized by acidic endosomes. This phenomenon indicates that H_2_S itself does not induce killing effect to the cancer cells, but clearly facilitates and amplifies the ferroptosis induced by Fe^2+^ ions released. In general, overexpression of CAT, an intrinsic characteristic of cancer cells, plays a vital role in initiation and progression of cancer and resistance to various therapies. The effect of H_2_S molecules on CAT activities of Huh7 cells and a comparison cell line (normal hepatocytes WRL‐68) was explored further. Interestingly, when the concentration of Na_2_S, the H_2_S donor, was increased, the activity of intracellular H_2_O_2_ enzyme was effectively suppressed in Huh7 cells, while that of normal cells barely changed (Figure 4g). Instead of Na_2_S, the inhibition effect on CAT activity of Huh7 cells was further confirmed by using FeS@BSA for cell culture for a short period (30 min), and a similar result was obtained, as expected (Figure S12, Supporting Information). Considering that CAT is highly associated with therapeutic resistance, a cell culture study using sorafenib‐resistant Huh7 cells was also pursued to demonstrate the strong therapeutic effect of FeS@BSA nanoclusters. As expected, FeS@BSA nanoclusters also presented effective inhibition effect on the viability of drug‐resistant cancer cells (Figure S13, Supporting Information), implying its therapeutic potential for drug‐resistant tumors.

Therefore, the mechanism of the strong in vitro anticancer effect presented by FeS@BSA nanoclusters is clear. As demonstrated in Figure 4h, after entering the endosomes and lysosomes of cancer cells through phagocytosis, rapid ionization of FeS@BSA nanoclusters occurs owing to the acid condition. Subsequently, both Fe^2+^ ions and H_2_S release, causing lysosome destabilization via proton sponge effect. In the cytoplasm, Fe^2+^ ions catalyze H_2_O_2_ to induce ·OH following the typical Fenton reaction. Meanwhile, the H_2_S gas released suppresses the activity of CAT (H_2_O_2_ enzyme) effectively, leading to the increased concentration of intracellular H_2_O_2_, which significantly facilitates iron‐based Fenton reaction and amplifies the ROS induction. It is noteworthy that the suppression effect of CAT by H_2_S may also weaken the therapeutic resistance of cancer cells to ROS.^[^[qv: 6,8]^]^ In consequence, the apoptosis of cancer cells is promoted by FeS@BSA nanoclusters.

The in vivo assessment of FeS@BSA nanoclusters was carried out using Huh7 tumor‐bearing nude mice. Mice with the tumors of almost 100 mm^3^ were used in our experiments with random division into four groups: 1) control group injected with PBS; 2) intratumoral injection of Na_2_S solution; 3) intravenous injection of Fe^2+^@BSA solution; and 4) intravenous injection of FeS@BSA solution. Injections were carried out once a week for 2 weeks, and the dosage for S^2−^ and Fe^2+^ for all the formulations was maintained equivalent for each injection. The in vivo MRI of Huh7 tumor‐bearing nude mice was performed at 0 (preintravenous), 1, and 2 h postintravenous administration of FeS@BSA nanoclusters. The T2‐weighted negative MRI signals in tumor tissue decreased significantly after the injection as revealed by the circled region in the MR images, indicating the high intratumoral accumulation of FeS@BSA nanoclusters (**Figure** **5**a).

**Figure 5:**
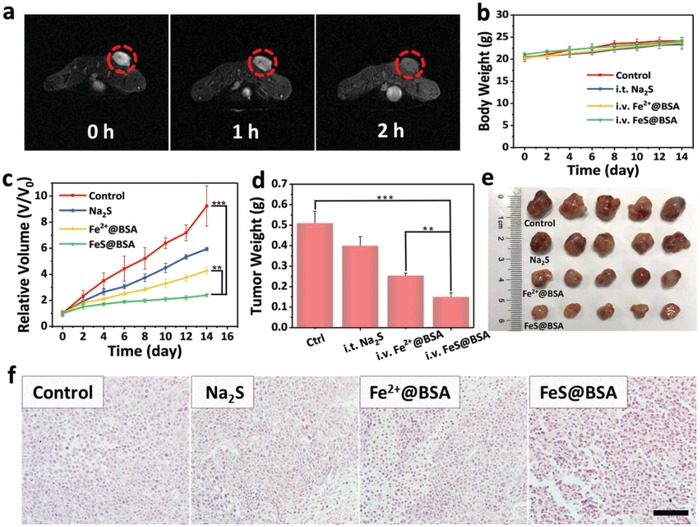
In vivo therapeutic properties. a) T2‐weighted MR images of Huh7 tumor‐bearing nude mice before and after the intravenous administration of FeS@BSA nanoclusters for 1 and 2 h. b) Body weight variation of the mice after different administration (*n* = 5, mean ± SD). c) Variation of tumor volume and d) tumor weight of the mice groups after various treatments (*n* = 5, mean ± SD). e) Photograph and f) images of H&E staining of the tumors extracted from the mice groups treated with different samples. Scale bar is 100 µm. ***p < 0.001, **p < 0.01, and *p < 0.05.

During the 2 weeks of treatment, no difference was observed in body weight among all groups. (Figure 5b). Mice treated with PBS showed fast‐growing tumors, and a certain inhibition was observed when the mice were injected with Na_2_S or Fe^2+^@BSA solutions. Na_2_S and Fe^2+^@BSA presented moderate inhibition rates at ≈27 and ≈50 wt%, respectively (Figure 5c,d). In contrast, tumor growth was remarkably suppressed in mice injected with the solution containing FeS@BSA nanoclusters. The highest tumor inhibition rate reached ≈71 wt%, indicating its excellent therapeutic properties in tumor suppression owing to the synergetic effect by the combination of Fe^2+^ and H_2_S. The representative photographs of mice and peeled tumor tissue agreed well to the findings (Figure 5e; Figure S14, Supporting Information). The histological examinations were carried out on tumor tissues collected from all four sample groups via hematoxylin and eosin (H&E) staining (Figure 5f) and Ki‐67 staining (Figure S15, Supporting Information). The tumor sections showed broadest area of necrosis and late‐stage apoptosis after treating with FeS@BSA solution, while the other two groups were partially destroyed, as expected.

Finally, the biosafety of FeS@BSA nanoclusters was further assessed systematically. After intravenous administration of FeS@BSA nanoclusters, the presence of Fe in blood, major organs (heart, liver, spleen, lung, and kidney), and tumor at different time points was examined by ICP‐MS (**Figure** **6**a,b). Owing to the low injection dose, the content of iron in plasma did not vary significantly, and the blood circulation half‐time of FeS@BSA was estimated to be 0.98 h. FeS@BSA nanoclusters mainly presented in the tumor, liver, and kidney after intravenous injection. The highest Fe accumulation in tumor was found to be at 2 h time point (from 52.8% to 82.1% i.d. g^–1^), which agreed with MRI results. The Fe presence in liver and kidney decreased as time proceeds mainly due to metabolism and excretion. In addition, the blood figures of mice were traced for up to 30 days (3, 7, and 30 days post‐injection) after intravenous injection with FeS@BSA solution at a high dose of 200 mg kg^−1^ (Figure 6c–i). Mice intravenously injected with PBS were used as the control group. Alanine aminotransferase (ALT), aspartate aminotransferase, direct bilirubin, total bilirubin, cholinesterase (CHE1), serum albumin (AlbG), and total protein were selected as the main parameters. It was shown that all blood figures maintained at a normal state compared with the control group. In addition, after 2 weeks of treatment, H&E staining images of major organs (heart, liver, spleen, lung, and kidney) from the mice indicated no clear variation occurred compared to the untreated mice (Figure 6j). The other two sample groups treated with Na_2_S and Fe^2+^@BSA solutions presented similar tissue characteristics (Figure S16, Supporting Information). All these findings suggest that FeS@BSA nanoclusters present expected in vivo compatibility to mice, implying its potential for clinical transformation.

**Figure 6:**
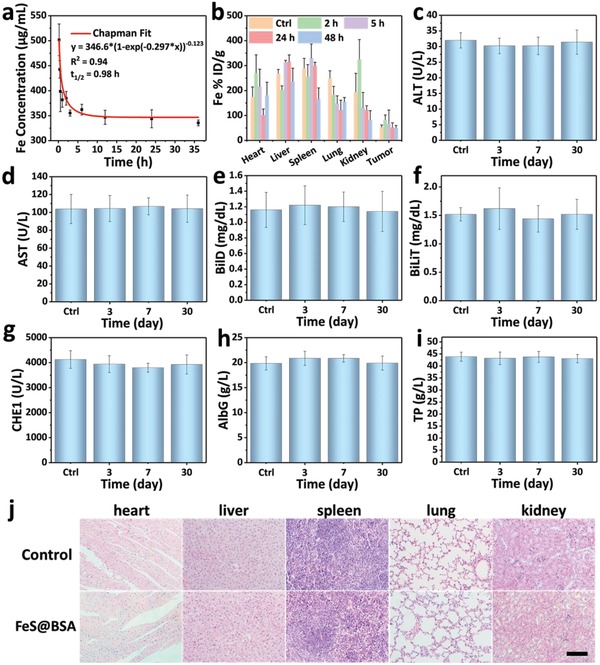
Biodistribution and biosafety evaluation of FeS@BSA nanoclusters. a) Fe concentration in the blood after intravenous injection of FeS@BSA at different time points (*n* = 3, mean ± SD). b) Biodistributions of Fe after intravenous administration at different time points (*n* = 3, mean ± SD). c–i) Blood figures of mice intravenously injected with 200 µL of FeS@BSA solution (20 mg mL^−1^), including alanine aminotransferase (ALT), aspartate aminotransferase (AST), direct bilirubin (BilD), total bilirubin (BiliT), cholinesterase (CHE1), serum albumin (AlbG), and total protein (TP), were recorded at 0, 3, 7, and 30 days (*n* = 3, mean ± SD). j) H&E‐stained slices obtained from the heart, liver, spleen, lung, and kidney of mice with and without intravenous FeS@BSA solution. Scale bar is 200 µm.

In summary, a new type of amorphous FeS@BSA nanoclusters were designed and synthesized via a self‐assembly approach for imaging‐guided synergetic tumor therapy. During the synthesis, Fe^2+^ and S^2−^ ions, which serve as the donor of ROS and H_2_S gas, respectively, were diffused into BSA nanoparticles to form FeS@BSA nanoclusters with tens of nanometers in dimension. BSA serves not only as the carrier matrix to ensure the colloid stability, but also as the protector for FeS compound from oxidation in the physiological environment. Owing to its amorphous nature, FeS@BSA nanoclusters may trigger rapid Fenton reaction to induce toxic ·OH, and to release H_2_S effectively within Huh7 cancer cells. After intravenous injection to the mice, the nanoclusters accumulated in tumor site through EPR effect and presented strong on‐site T2‐weighted MRI signals. More importantly, the intracellular H_2_S induced effective inhibition to the CAT activity specifically in the Huh7 cancer cells, and in turn promoted the intracellular content of H_2_O_2_ and amplified the production for toxic ROS significantly. In consequence, both in vitro and in vivo anti‐tumor efficacy were enhanced by a large magnitude, while no clear side effects to the main organs and blood figures of mice were observed. This gas‐amplified ROS‐based therapeutic system has, therefore, offered another way of thinking in the current exploration of the nanoplatforms for cancer treatment with high efficacy as well as the potential for clinical transformation.

## Conflict of Interest

The authors declare no conflict of interest.

## Supporting information

Supporting Information
