# Supplementary material for: FeS@BSA Nanoclusters to Enable H2S‐Amplified ROS‐Based Therapy with MRI Guidance
Source: Adv Sci (Weinh). 2020 Feb 19;7(7):1903512. doi: 10.1002/advs.201903512 (PMC7141047; doi:10.1002/advs.201903512)
Supplement: Supplementary file 1 — Supporting Information [file ADVS-7-1903512-s001.pdf]

Copyright WILEY-VCH Verlag GmbH & Co. KGaA, 69469 Weinheim, Germany, 2020.

# Supporting Information

for *Adv. Sci.,* DOI: 10.1002/advs.201903512

# FeS@BSA Nanoclusters to Enable $H_2S$-Amplified ROS-Based Therapy with MRI Guidance

*Congkun Xie, Dong Cen, Zhaohui Ren, Yifan Wang, Yongjun Wu, Xiang Li,* Gaorong Han,* and Xiujun Cai**

# Supporting Information

## FeS@BSA Nanoclusters to Enable $H_2S$ Amplified ROS-based Therapy with MRI Guidance

Congkun Xie [1,¤], *Dong Cen*[2,¤], *Zhaohui Ren*[1], *Yifan Wang*[2], *Yongjun Wu*[1], *Xiang Li* [1,]*, *Gaorong Han* [1,] *, *Xiujun Cai* [2,]*

[1] State Key Laboratory of Silicon Materials, School of Materials Science and Engineering, Zhejiang University, Hangzhou, Zhejiang 310027, P. R. China
[2] Key Laboratory of Endoscopic Technique Research of Zhejiang Province, Sir Run Run Shaw Hospital, Zhejiang University, Hangzhou 215123, P. R. China

* Corresponding Author: xiang.li@zju.edu.cn (XL); hgr@zju.edu.cn (GRH); srrsh_cxj@zju.edu.cn (XJC)
¤ Authors with equal contribution

### Experimental Section

*Materials*: Bovine serum albumin (BSA) was purchased from Sangon Biotech (Shanghai) Co., Ltd. Ferrous chloride tetrahydrate, N,N-dimethyl-p-phenylenediamine dihydrochloride, 5,5-Dimethyl-1-pyrroline N-oxide (DMPO) and DCFH-DA (≥97%) were obtained from Sigma-Aldrich. Sodium sulfide nonahydrate was purchased from Macklin. Zinc acetate, ferric trichloride, 1,3-Diphenylisobenzofuran (DPBF) was purchased from Aladdin Co., Ltd. Sodium acetate anhydrous was obtained from Sinopharm Chemical Reagent Co., Ltd. Catalase Assay Kit was obtained from Beyotime® Biotechnology. WSP-1 ($H_2S$ probe) was purchased from Shanghai Maokang Bio Co., Ltd. All regents were used as received.

*Synthesis of FeS@BSA nanoclusters*: 50.0 mg BSA was dissolved in 6.0 mL of deionized water, 1.0 mL of $FeCl_2$ solution (37 mM) was added under vigorous stirring, followed by the addition of 1.5 mL of $Na_2S$ solution (37 mM). Then, the mixture was allowed to react at 4 °C for 12 h. FeS@BSA nanoclusters were obtained after dialysis (MW: 8000-14000) against deionized water for 12 h at 4 °C, and freeze-dried for the further use.

As a comparison, $Fe^{2+}$ ions contained BSA nanoparticles ($Fe^{2+}$@BSA) were prepared following the similar procedure of FeS@BSA nanoclusters, except replacing $Na_2S$ solution with equal volume of deionized water. Furthermore, crystalline FeS nanoparticles were also synthesized according to a typical approach reported previously.[1] In brief, 0.225 g thioacetamide, 1.176 g ammonium ferrous sulfate and 0.729 g ammonium citrate were added into 30 mL water under stirring. After removing oxygen from the solution by $N_2$ bubbling, the solution was placed in oil bath at 85 °C for 6 h. The final crystalline FeS was obtained by centrifugation, and washed with water and ethanol for several times.

*Characterization*: The microstructure of samples was examined using a field-emission scanning electron microscopy (FESEM, Hitachi SU-70) and transmission electron microscopy (TEM, Tecnai F20, FEI). The hydrodynamic size was determined by dynamic light scattering (DLS) using Zetasizer (Zetasizer Nano-ZS, Malvern). The freeze-dried powder sample was characterised via an X-ray diffraction (XRD) on a X'pert PRO MPD, scanning 2θ from 10° to 80°. The CD spectra were measured by CD Spectropolarimeter (Jasco-1500), and the UV-vis spectra were recorded by a TU-1810 UV-vis spectrophotometer. X-ray photoelectronspectroscopy (XPS) was performed on a AXIS Supra, Kratos.

*Exploration of pH-triggered $H_2S$ release phenomenon*: FeS@BSA nanoclusters were dispersed in PBS solutions with varied pH (7.4, 6.5 and 5.5) under gentle shaking at 37 ℃. The concentration was set at 1 mg $mL^{-1}$. Subsequently, 2 mL FeS@BSA solution was collected at the time intervals of 0.2, 0.5, 1, 2, 3, 4, 6, 8, 24, 36, 48 and 72 h. The unreacted FeS@BSA nanoclusters were removed by ultrafiltration to avoid the possible interference for $H_2S$ measurement. The concentration of $H_2S$ was measured with a standard method as described previously.[2] Briefly, 1.5 mL of solution was mixed with zinc acetate/sodium

acetate mixture (4:1 mass ratio, 1 mL). Methylene blue was then formed by the addition of N, N-dimethyl-p-phenylenediamine dihydrochloride and $FeCl_3$. After incubation for 15 minutes, the absorbance at 665 nm was examined, and the concentration of $H_2S$ was determined using a standard curve of $Na_2S$ (Figure S17, Supporting Information).

*Examination of hydroxyl radicals (·OH) induction*: 1,3-diphenylisobenzofuran (DPBF) was used to examine the ·OH production.[3] Briefly, DPBF in DMSO was mixed with FeS@BSA nanoclusters and $H_2O_2$ solution, and then the absorbance at 410 nm was measured with an UV-vis spectrophotometer. Varied concentrations of FeS@BSA nanoclusters (containing 1.7, 2.5, 3.3 μg $mL^{-1}$ FeS respectively), $H_2O_2$ (100, 150, 200 μM) and different pHs (pH = 7, 6.5 and 6) were used, respectively, to investigate the effect of pH, FeS@BSA concentration and $H_2O_2$ on the ·OH induction.

*In vitro and In vivo MR Imaging*: For in vitro MRI performance, FeS@BSA solutions with different concentrations (Fe concentration: 0.5, 1, 2, 3, 4 mM) were imaged by a 3.0 T clinical MR scanner. For in vivo MR imaging, mice were intravenously injected with 200 μL of FeS@BSA solution, and imaged by a 3.0 T clinical MR scanner after 0 h (pre-), 1 h and 2 h injection.

*In vitro cytotoxicity assays*: WRL-68 normal cells and Huh7 cancer cells were cultured in Dulbecco's modified Eagle's medium containing 10% fetal bovine serum and 1% penicillin and streptomycin in a 37 °C incubator with 5% $CO_2$. Cell Counting Kit-8 (CCK-8) assay was used to determine the cell viability. Typically, cells (5000 cells/well) were seeded in 96-well plates and incubated at 37 °C for 24 h. Then the medium was replaced with fresh culture medium containing FeS@BSA nanoclusters at different concentrations. After incubation for 24 or 48 h, the medium was discarded and CCK-8 solution was added to each well.

Incubating for 1-4 hours in the incubator and mixing gently on an orbital shaker for 1 minute, the absorbance at 450 nm was measured using a microplate reader (Tecan 50, The Switzerland).

Furthermore, live/dead cell staining was also conducted to evaluate cytotoxicity. Briefly, cells were cultured in 6-well plate for 24 h, and then treated with PBS, $Na_2S$, $Fe^{2+}$@BSA and FeS@BSA respectively and further cultured for 24 h. Subsequently, the cells were stained with Calcein-AM/PI for 15 min, washed twice with PBS and observed under fluorescent microscope (Nexcope, USA).

*Intracellular ·OH detection*: After incubating Huh7 cells in 6-well plates with FeS@BSA nanoclusters (20 μg $mL^{-1}$) in neutral and acidic (pH = 6) medium respectively for 6 h, the cells were washed twice with PBS, and then 2 mL of PBS and DCFH-DA (20 μM) were added. After incubation for further 20 min, the cells were observed under fluorescent microscope (Ex/Em: 485/525 nm). The cells incubated without treatment of FeS@BSA were used as the blank control.

*Intracellular $H_2S$ detection*: WSP-1 was used as intracellular $H_2S$ probe. Briefly, after treatment of cells with FeS@BSA nanoclusters (pH = 7.4 and pH = 6.0) for 6 h (PBS for control), WSP-1 (15 μM) was added, followed by incubation for 30 min. Subsequently, the cells were washed twice with PBS and observed under fluorescent microscope (Ex/Em: 465/515 nm).

*Detection of catalase (CAT, $H_2O_2$ enzyme) activity*: CAT activity was determined by using Catalase Assay Kit. Typically, cells were collected and suspended in PBS ($1 \times 10^6$ $mL^{-1}$). Subsequently, 2 mL of the cell suspension was treated with $Na_2S$ solutions (0.4, 0.8, 1.2, 1.6

mM, respectively), and the control group was treated with PBS only. After 30 minutes, the cells were centrifuged and washed for 3 times, and cell lysate was collected by cell lysis buffer for CAT activity detection. The later procedure was according to kit protocol, and relative enzyme activity was calculated as follows: absorbance in each group/ absorbance of control group. Similarly, for examining the effect of $H_2S$ released from FeS@BSA on catalase activity, WRL-68 and Huh7 cells were incubated in a mild acidic culture medium (pH 6.0) containing FeS@BSA (0.1 mg $mL^{-1}$), and that without treatment of FeS@BSA was set as control. After 30 min, CAT activities were detected.

*In vivo study*: 4-6 weeks old male BALB/c nude mice were purchased from Shanghai Laboratory Animal Center, and all animal experiments were approved by the Ethics Committee of Sir Run Run Shaw Hospital. To develop the tumor model, Huh7 cells ($5 \times 10^6$) in PBS (200 μL) were subcutaneously injected into the right side back of each mouse. When the tumor volume grew up to ~150 $mm^3$, the mice were randomly divided into four groups with following treatments: (1) control injected with saline, (2) intratumoral injection of $Na_2S$, (3) intravenous injection of $Fe^{2+}$@BSA, and (4) intravenous injection of FeS@BSA. Injections were carried out once a day for 10 days, and the dosage for $S^{2-}$ and $Fe^{2+}$ of all the formulations was equivalent for each injection (7 mg $kg^{-1}$ FeS). The tumor volume and body weight were measured every day. Tumor volume was calculated according to the following formula: $width^2 \times length/2$. After the treatment course, all mice were sacrificed and tumors were peeled for histopathological analysis including H&E staining and Ki-67 staining.

*Bio-safety assessement*: For histopathological analyses of major organs, the mice were sacrificed after the treatment to collect the major organs (heart, liver, spleen, lung and kidney). The tissue samples were fixed in 4% paraformaldehyde solution and stained with hematoxylin and eosin (H&E). For blood analysis, healthy mice were intravenously injected with 200 μL

of FeS@BSA solution (10 mg $mL^{-1}$) and sacrificed at different time intervals after injection (3, 7 and 30 d, 6 mice at each interval), and healthy mice without injection of FeS@BSA nanoclusters were used as the control group. Subsequently, whole blood was collected and examined in sir run run shaw hospital. The recorded blood figures included alanine aminotransferase (ALT), aspartate aminotransferase (AST), direct Bilirubin (BilD), total bilirubin (BiliT), cholinesterase (CHE1), serum albumin (AlbG) and total protein (TP). For blood circulation assay, 20 μL blood sample was collected from inner canthus of every three mice at different time points (0.17, 0.33, 0.5, 1, 2, 3, 6, 12, 24, 36 h) after intravenous injection, and dissolved in 0.5 mL heparin sodium solution (50 units $mL^{-1}$). Subsequently, the samples were digested in nitric acid/perchloric acid (9:1) at 260 °C. The concentration of Fe was measured by ICP-MS. Similarly, major organs (heart, liver, spleen, lung and kidney) and tumor (n = 3) were harvested at time points of 2, 5, 24 and 48 h for biodistribution assessment.

**Supporting Figures**

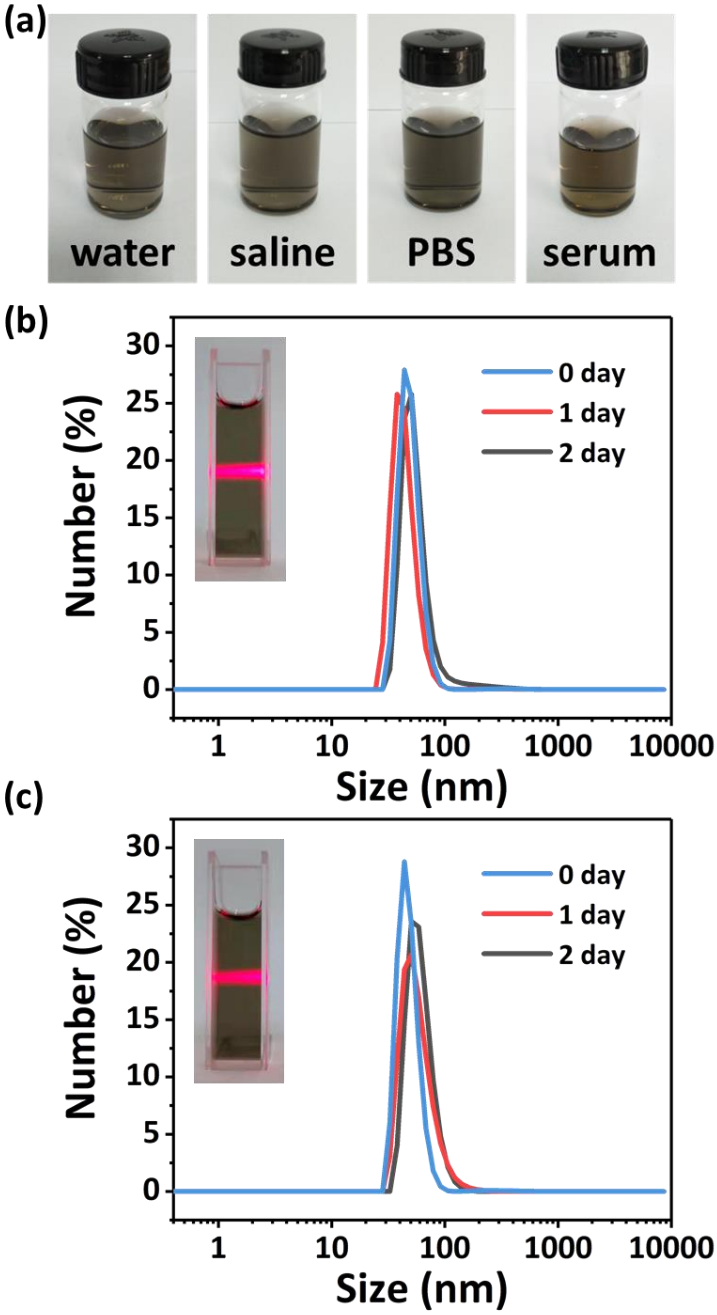


**Figure S1. Colloidal stability of FeS@BSA.** (a) The optical images of FeS@BSA solutions with pure water, normal saline, PBS and serum after 2 days. Tyndall effect and DLS profiles of FeS@BSA nanoclusters for 2 days in (b) pure water and (c) normal saline. The results demonstrated that FeS@BSA nanoclusters possessed good colloidal stability in all solutions.

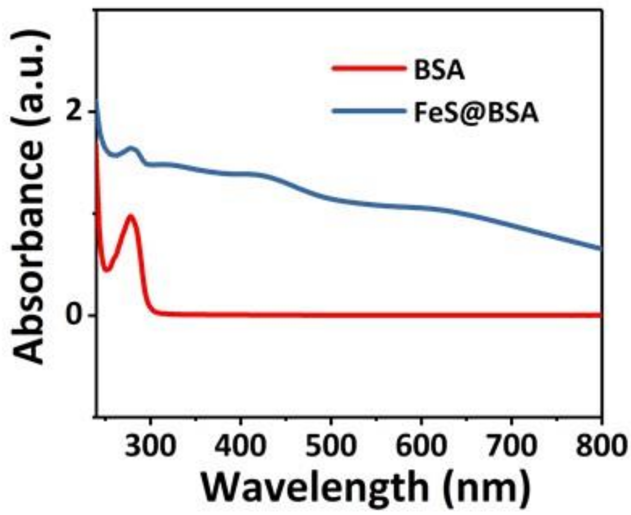


**Figure S2. UV-vis spectra of pure BSA and FeS@BSA.** FeS@BSA nanoclusters showed typical BSA absorption peak at ~280 nm.

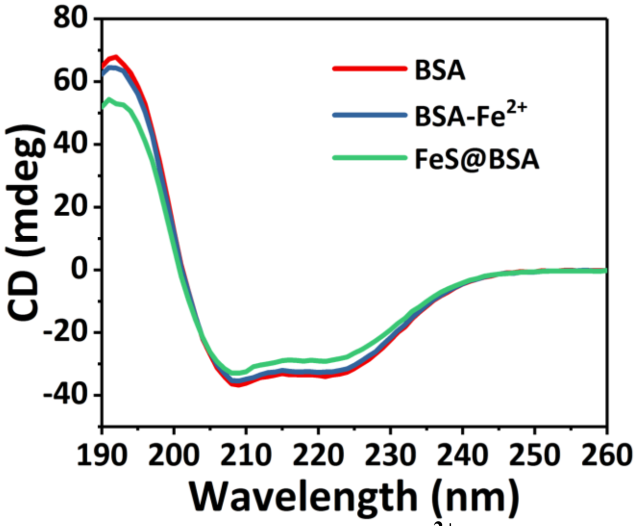


**Figure S3. The CD spectra of pure BSA, BSA-$Fe^{2+}$ and FeS@BSA.** The CD spectra of BSA-$Fe^{2+}$ and FeS@BSA presented no clear variation compared with that of pure BSA, implying that the secondary structure of BSA did not change significantly during the synthesis of FeS@BSA.

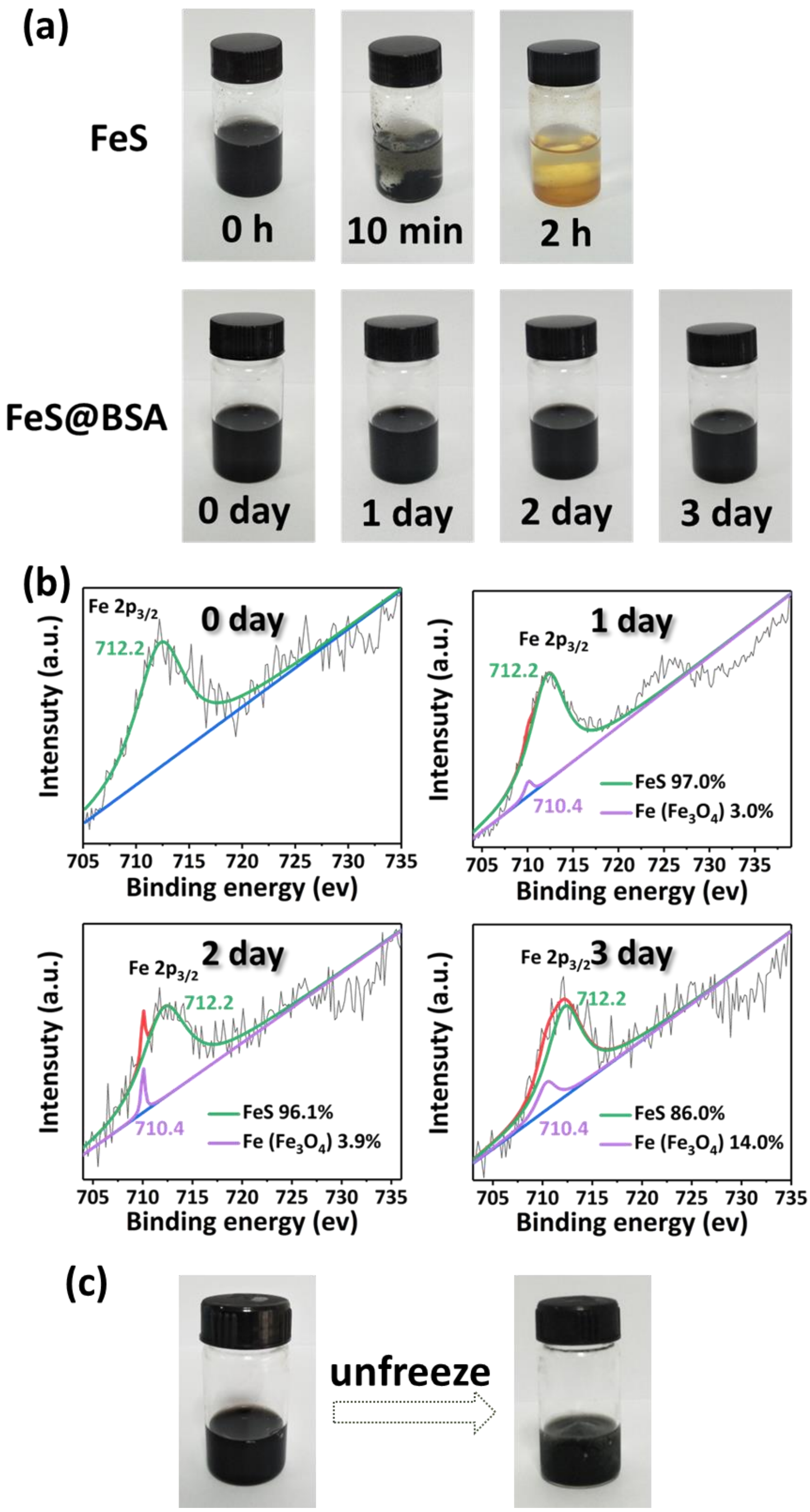


**Figure S4. The protection of FeS from oxidation by BSA matrix.** (a) FeS nanoparticles in water solution precipitates and be oxidized in 2 hours, while FeS@BSA solution remains stable for 3 days. (b) Corresponding XPS spectra of FeS@BSA freshly prepared and stored for 1, 2 and 3 days. (c) FeS@BSA solution remains stable after being freezed and unfreezed.

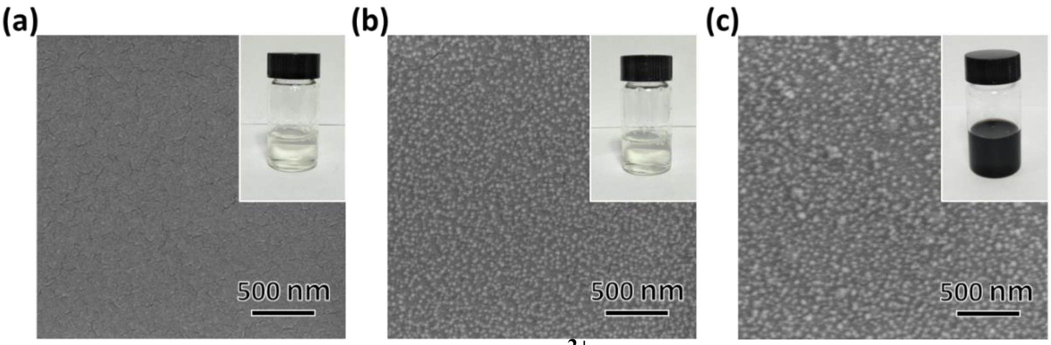


**Figure S5. Morphology of (a) BSA, (b) BSA-$Fe^{2+}$ and (c) FeS@BSA nanoparticles.** Pure BSA does not present clear particulate morphology, while nanoparticles reform after adding $Fe^{2+}$ into BSA solution. No obvious variation is observed during the process of FeS@BSA formation after adding $S^{2-}$, but the color of solution turns into black. Inset: Photos of the corresponding solutions.

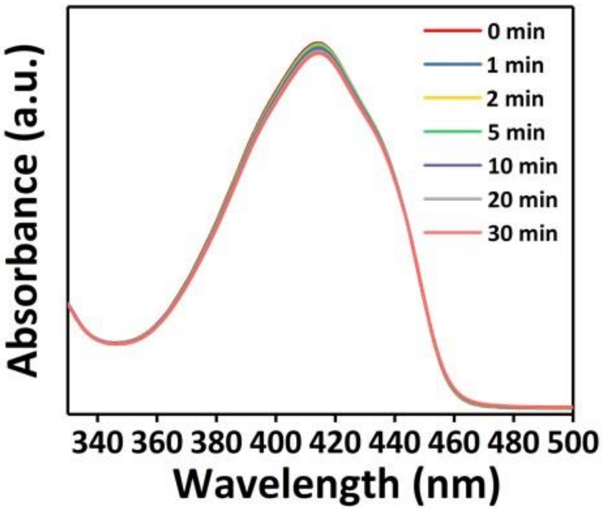


**Figure S6. UV-vis spectra of DPBF solution with pH of 6 and 200 μM $H_2O_2$ after being stored for different period of time.**

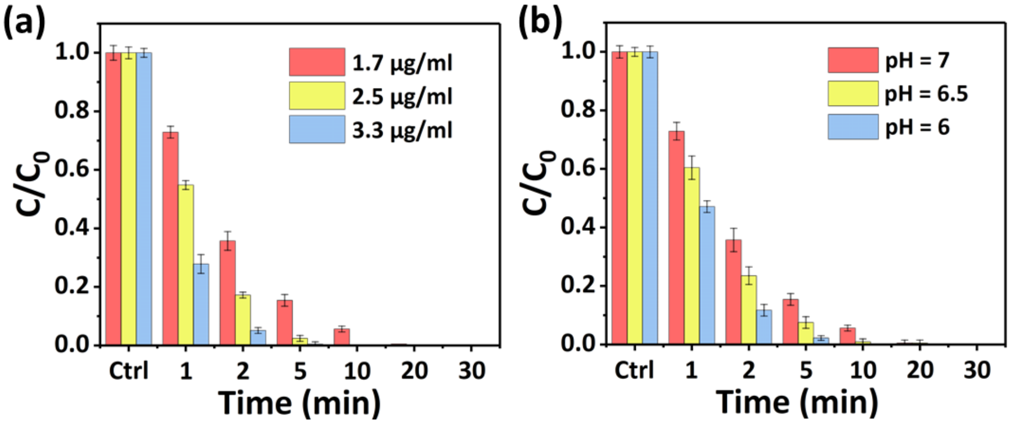


**Figure S7. DPBF degradation examinations with (a) different concentrations of FeS@BSA (pH = 7 with 150 μM $H_2O_2$) and (b) different pHs (1.7 μg/mL FeS@BSA with**

**150 μM $H_2O_2$) (n = 3, mean ± s.d.).** It demonstrates that higher FeS@BSA concentration and lower solution pH may induce rapid ·OH production.

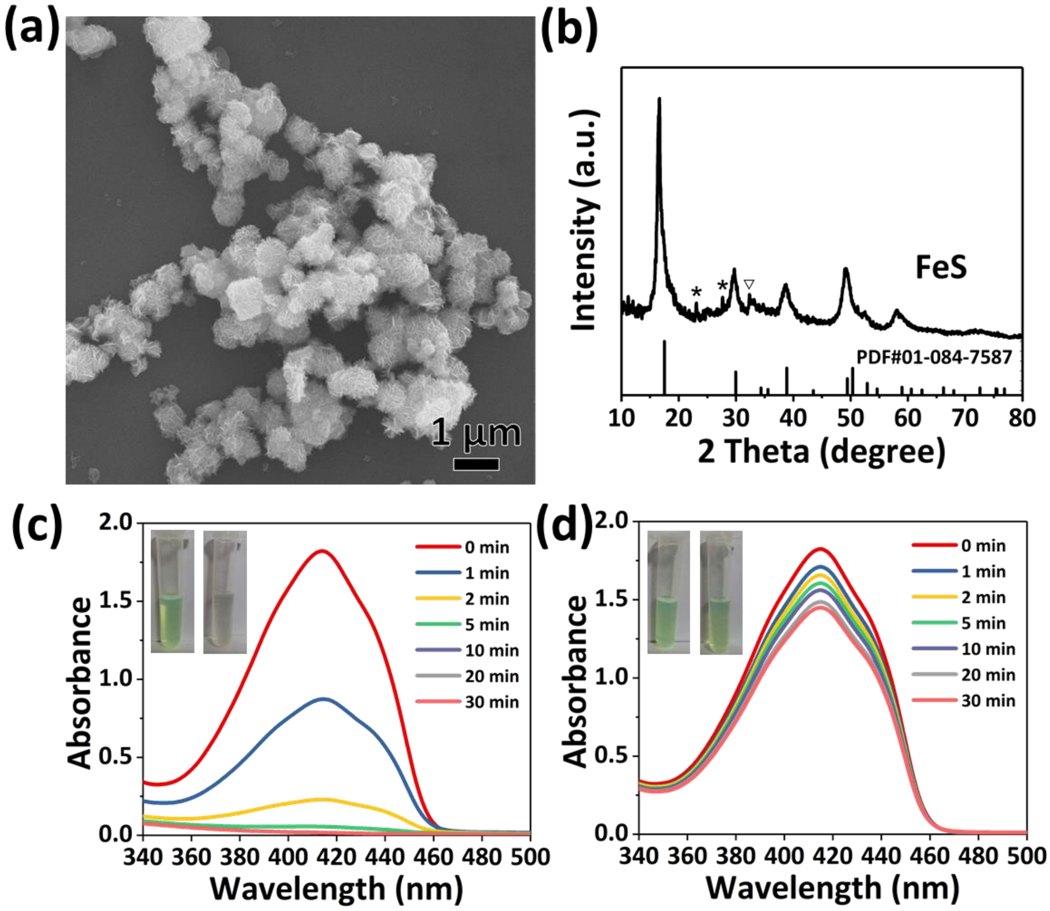


**Figure S8. Examination of ROS induction by crystalline FeS particles using DPBF.** (a) SEM and (b) XRD of crystalline FeS particles (The diffraction peaks marked with  and * result from $Fe_2O_3$ and S, respectively, originating from partial oxidation of FeS). The degradation of DPBF solutions containing (c) FeS@BSA nanoclusters and (d) crystalline FeS at $H_2O_2$ concentration of 150 μM and pH = 6. Inset: Photos of the DPBF before and after degradation for 30 minutes.

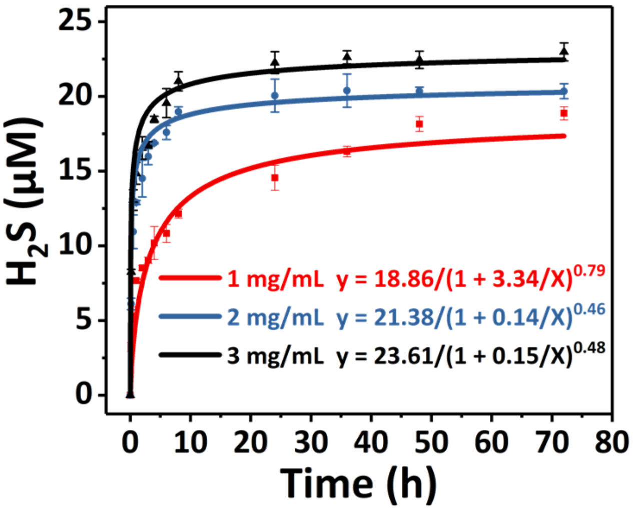


**Figure S9. $H_2S$ release profile in pH 5.5 with different concentrations of FeS@BSA nanoclusters (n = 3, mean ± s.d.).**

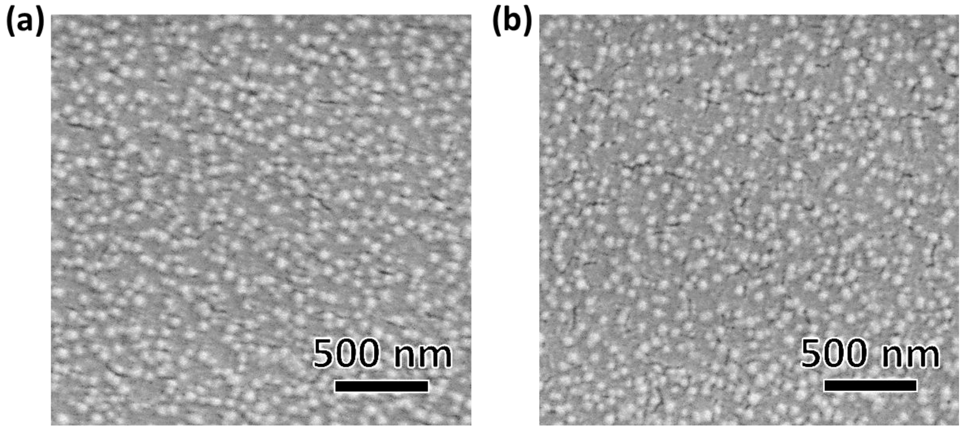


**Figure S10. SEM images of FeS@BSA (a) before and (b) after $H_2S$ releasing.** The morphology of FeS@BSA clusters do not present clear variation after $H_2S$ gas release.

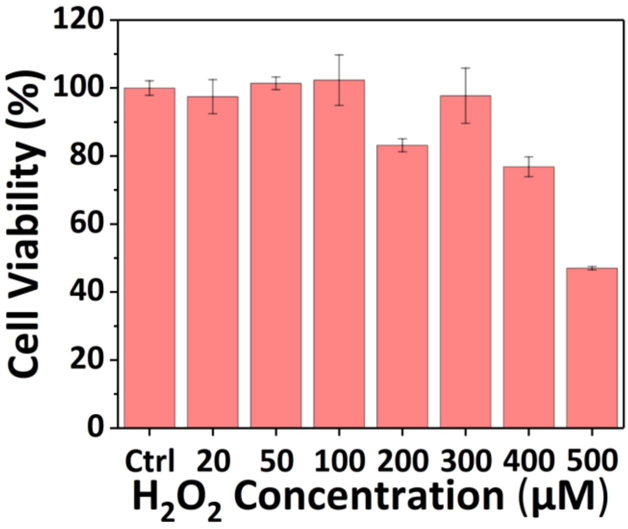

**Figure S11. Cell viability of Huh7 cultured with $H_2O_2$ for 24 h (n = 3, mean ± s.d.).** There is no toxic effect on cell proliferation when incubation with $H_2O_2$ at concentration of 100 μM.

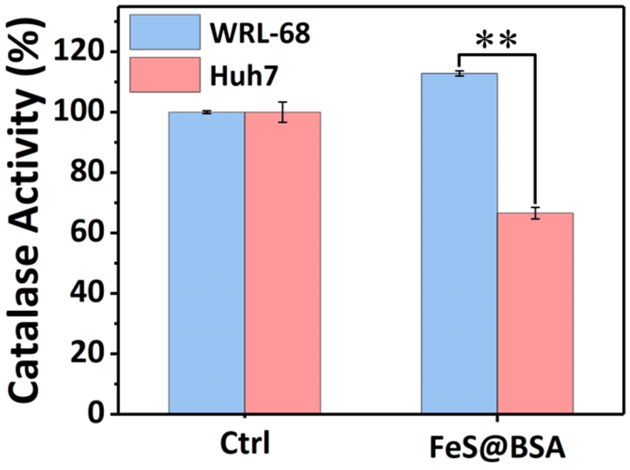


**Figure S12. Effect of $H_2S$ released from FeS@BSA (0.1mg $mL^{-1}$) on catalase activity of WRL-68 and Huh7 cells (n = 3, mean ± s.d., **p < 0.01).**

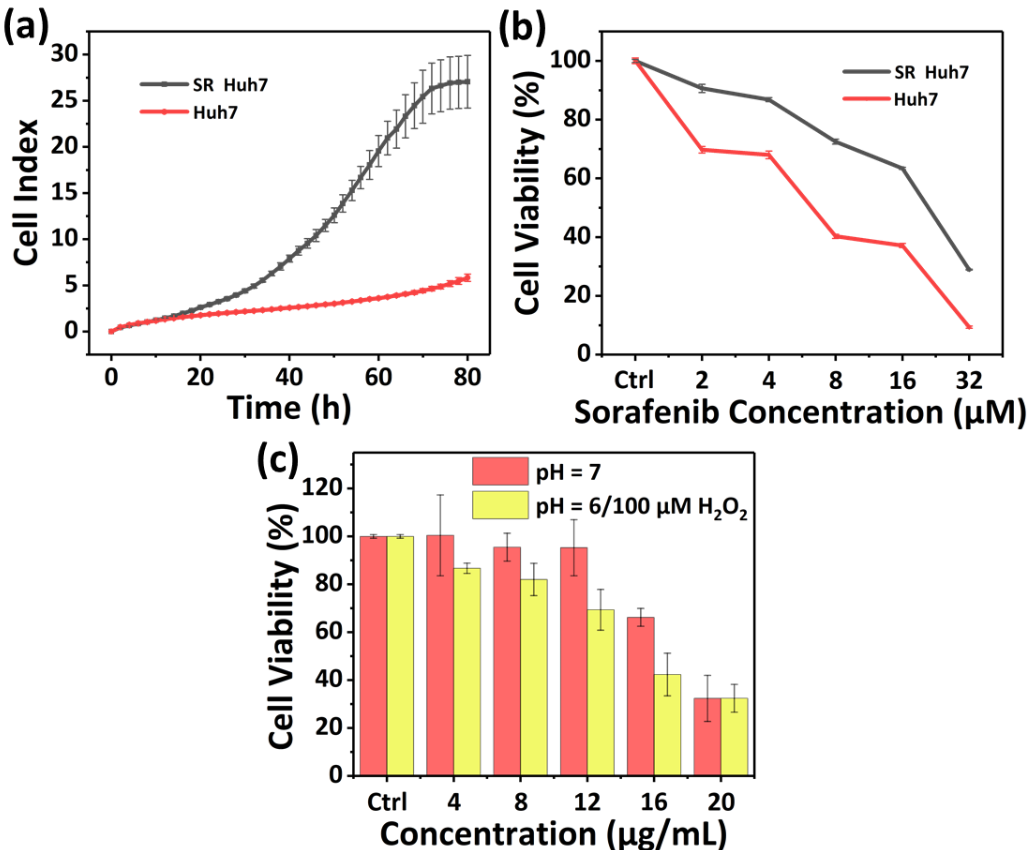


**Figure S13. Cell-killing effect of FeS@BSA nanoclusters on Sorafenib resistant (SR) Huh7 cells.** (a) Cell index of SR Huh7 and Huh7 cultured with Sorafenib. (b) Cell viability of SR Huh7 and Huh7 after cultured with Sorafenib at different concentrations for 24 h. (c) Viability of SR Huh7 cultured with FeS@BSA solutions with different concentrations and

stated conditions after 24 h (n = 3, mean ± s.d.).

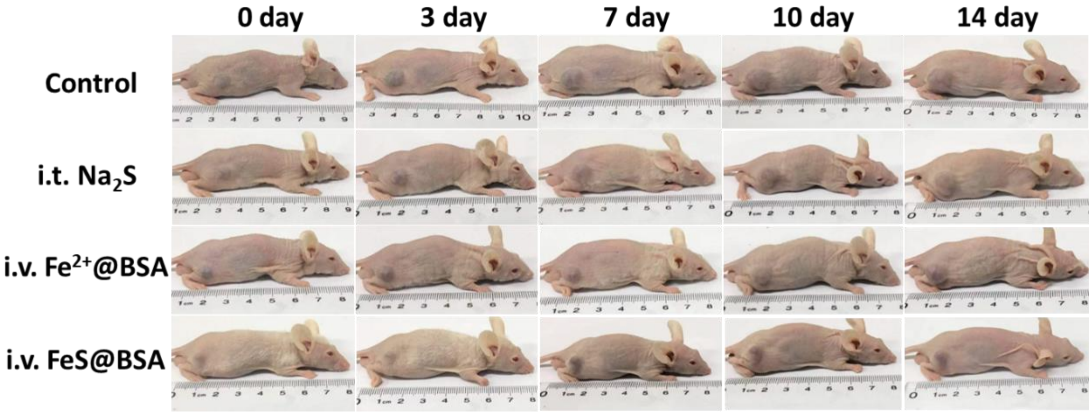


**Figure S14. Representative photographs of each mice group.** In vivo therapeutic effect of FeS@BSA group is superior than that of other groups.

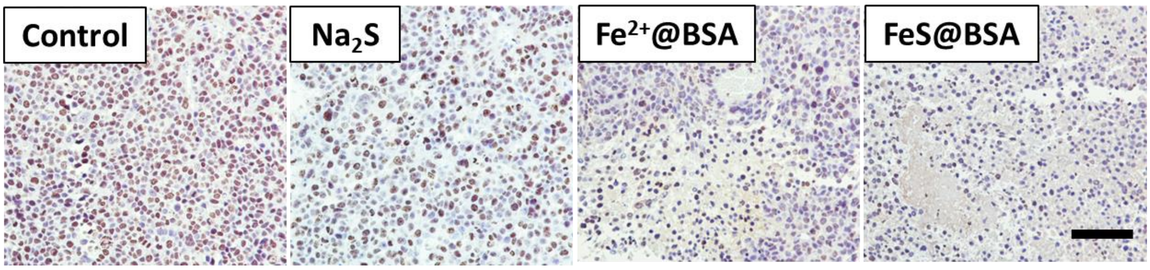


**Figure S15. Ki-67 stained images of tumor slices collected after receiving different treatments for 2 days**. Compared with the control group, only marginal decrease in positive nucleus (dark brown) occurred in $Na_2S$ and $Fe^{2+}$@BSA treatment groups, while FeS@BSA treatment group showed the most dramatic decrease of positive nucleus, indicating the strongest suppression effect on cell proliferation. Scale bar is 100 μm.

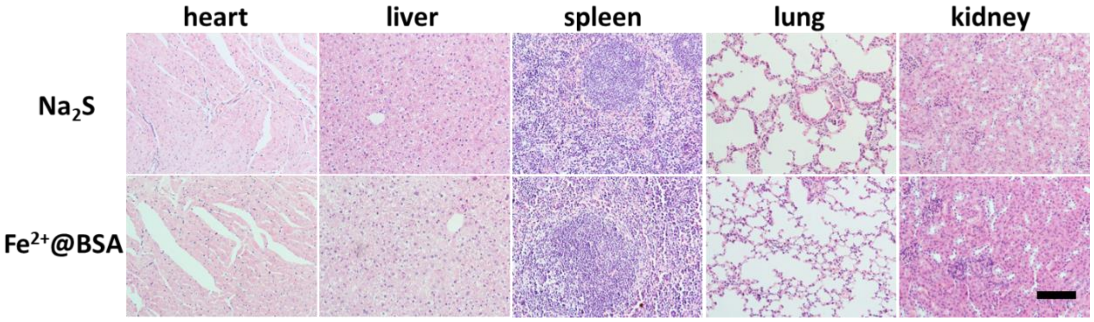


**Figure S16. H&E stained images of the major organs (heart, liver, spleen, lung and kidney) of mice collected 2 days after receiving $Na_2S$ and $Fe^{2+}$@BSA treatment.** No clear tissue damage or inflammatory lesion was observed in these major organs, demonstrating low side effect after $Na_2S$ and $Fe^{2+}$@BSA treatments. Scale bar is 200 μm.

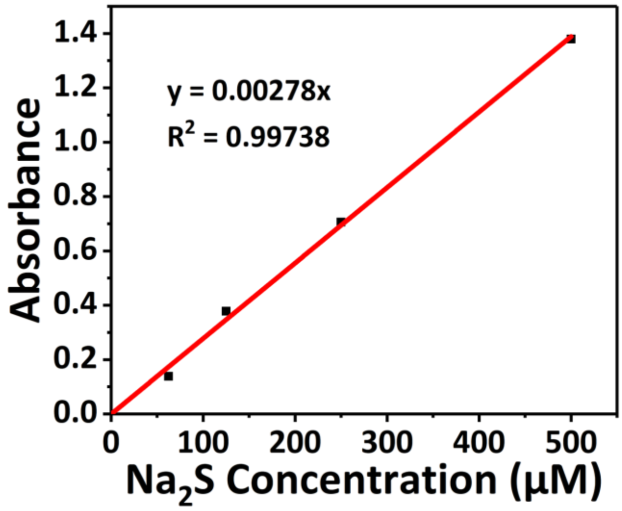


**Figure S17. $H_2S$ standard curve.**

**Reference**

[1] Z. Wang, L. Wu, M. Chen, S. Zhou, *J. Am. Chem. Soc.* **2009**, 131, 11276.
[2] Z. W. Lee, J. Zhou, C.-S. Chen, Y. Zhao, C.-H. Tan, L. Li, P. K. Moore, L.-W. Deng, *Plos One* **2011**, 6.
[3] C. Fang, P. Yan, Z. Ren, Y. Wang, X. Cai, X. Li, G. Han, *Applied Materials Today* **2019**, 15, 472.
